# Carotid Artery: A Window for the Assessment of Multiple Cardiovascular Risks

**DOI:** 10.31662/jmaj.2021-0024

**Published:** 2021-03-26

**Authors:** Hirofumi Tomiyama

**Affiliations:** 1 Department of Cardiology, Tokyo Medical University, Tokyo, Japan

**Keywords:** Atherosclerosis, cardiovascular disease, arterial stiffness

Several cardiovascular risk assessment models that calculate conventional risk factors, such as Framingham risk score or Suita score, are available. Although these risk models are simple and useful, under- or overestimations of cardiovascular risks are noted in some cases. Therefore, secondary tools that validate these models for cardiovascular risk assessment are needed. Atherosclerotic vascular damages cause morphological changes in arterial trees, such as vascular hypertrophy, enlargement, stenosis, and/or torsion ^(1)^. Furthermore, the most important function of arterial tree is constantly supplying blood to peripheral organs, and media layers contribute to generate continuous blood flow via its cushion effect, but atherosclerotic vascular damage can impair this function by increasing arterial stiffness ^(1), (2), (3)^. Morphological assessment of atherosclerotic vascular damage using ultrasound examination and functional assessment of vascular damage using methods such as the assessment of endothelial function, pulse wave velocity, and pulse wave analysis have been proposed as secondary tools and have already been applied in the clinical setting ^(1), (2), (3)^. A morphological assessment of vascular damage may reflect the cardiovascular risk associated with atherosclerotic plaque and its vulnerability, whereas carotid artery ultrasound examination can detect carotid arterial stenosis in a noninvasive manner. Conversely, functional assessment may reflect the cardiovascular risks related to abnormal cardiovascular hemodynamics, such as increased cardiac afterload, impaired coronary blood supply, microvascular damage, etc ^(3)^. Thus, both tools reflect different facets of cardiovascular risk and may be used as individual markers for cardiovascular risk.

Vascular damage can be assessed using various vascular beds. Morphological assessments are conducted in the carotid artery, thoracic and abdominal aorta, or femoral artery. In functional assessment, the endothelial function of the finger or brachial arteries is assessed; pulse wave velocity is measured at upper extremities, lower extremities, or aorta; and pulse wave analysis is conducted for the radial artery or carotid artery. Sam et al. proposed that the carotid artery is a useful window in the assessment of multiple risks, i.e., functional as well as morphological assessment of atherosclerotic vascular damage ^(4)^. In the morphological assessment of the carotid artery, three dimensional carotid plaque is assessed in 2 dimensions. Using carotid pulse wave analysis, the applanation tonometry of the radial or carotid arteries to measure pressure wave forms can be technically and anatomically challenging, and its measurement still lacks accuracy. Furthermore, this technique requires the operator to apply a mild hold-down pressure to flatten the arterial wall and obtain the optimal applanation as a stable baseline ^(5)^. Because of these methodological limitations, the generality and reproducibility of the measurement of carotid intima-media thickness and plaque and of carotid pulse wave analysis have not been fully established. Therefore, further developments are needed to apply the carotid artery as a simple window to assess multiple cardiovascular risks.

## Article Information

### Conflicts of Interest

None
